# Short-term effects of Vertical sleeve gastrectomy and Roux-en-Y gastric bypass on glucose homeostasis

**DOI:** 10.1038/s41598-019-51347-x

**Published:** 2019-10-15

**Authors:** Oddrun Anita Gudbrandsen, Simon Nitter Dankel, Lillian Skumsnes, Tone Nygaard Flølo, Oddry Henriette Folkestad, Hans Jørgen Nielsen, Villy Våge, Arne Christian Mohn, Bjørn Gunnar Nedrebø, Jørn V. Sagen, Johan Fernø, Gunnar Mellgren

**Affiliations:** 1 Department of Clinical Medicine, University of Bergen, N-5020 Bergen, Norway; 2 Mohn Nutrition Research Laboratory, Department of Clinical Science, University of Bergen, N-5020 Bergen, Norway; 3 Hormone Laboratory, Haukeland University Hospital, N-5021 Bergen, Norway; 4 Surgical Department, Haugesund Hospital, N-5504 Haugesund, Norway; 5 Department of Surgery, Voss Hospital, Haukeland University Hospital, N-5704 Voss, Norway; 6 Department of Clinical Science, University of Bergen, N-5020 Bergen, Norway

**Keywords:** Medical research, Diabetes

## Abstract

The objective of this study was to compare the biochemical changes related to glucose tolerance and lipid metabolism in non-diabetic patients shortly after vertical sleeve gastrectomy (SG) or Roux-en-Y gastric bypass (RYGB). Non-diabetic women and men with morbid obesity were studied the day before and six days after SG (N = 15) or RYGB (N = 16). Patients completed an oral glucose tolerance test (OGTT; 75 g glucose) at both visits. SG and RYGB similarly improved fasting glucose homeostasis six days after surgery, with reduced glucose and insulin concentrations. The OGTT revealed differences between the two surgery groups that were not evident from the fasting serum concentrations. Postprandial (120 min) glucose and insulin concentrations were lower after RYGB but not after SG, whereas concentrations of glucagon-like peptide-1, peptide YY, glucagon and non-esterified fatty acids were elevated after both SG and RYGB. Fasting triacylglycerol concentration did not change after surgery, but concentrations of high density lipoprotein and low density lipoprotein cholesterols were reduced in both surgery groups, with no differences between the groups. To conclude, RYGB induced a more pronounced improvement in postprandial glucose homeostasis relative to SG, possibly due to improved insulin sensitivity rather than augmented insulin concentration.

## Introduction

The prevalence of obesity has increased in recent decades, and obesity is now one of the leading public health concerns worldwide. The most effective and recognized method for treating morbid obesity is bariatric surgery^1–3^. Most patients undergoing bariatric surgery have reduced insulin sensitivity, 30% have prediabetes and a smaller proportion of approximately 20% of the patients have type 2 diabetes (T2D)^4^. Glucose concentrations often normalize within a few days after bariatric surgery, i.e. before significant weight loss is achieved^5^, and one explanation may be changes in hormone secretion as a result of intestinal/gut rearrangement, such as increased glucagon like peptide 1 (GLP-1). In most studies on patients undergoing bariatric surgery, the analyses of endocrine signals with potential effect on glucose homeostasis are performed after significant weight reduction has occurred, and it is therefore difficult to establish whether the observed effects on glucose and insulin concentrations are entirely consequences of weight reduction.

In the present study we compared the short-term (six days) effects of vertical sleeve gastrectomy (SG) and Roux-en-Y gastric bypass (RYGB) performed in non-diabetic patients with severe obesity. SG and RYGB are presently the most commonly performed bariatric procedures worldwide^6^. SG results in similar weight loss but less improvement in T2D and hyperlipidemia as RYGB after 24 months^7^. The short-term effects of RYGB and SG have been investigated by others, demonstrating larger increase in GLP-1 and insulin concentrations after RYGB relative to SG, after a liquid meal 8–10 days postoperatively^8^ in a mixed group of diabetic and non-diabetic patients. Additional well-controlled studies in homogenous groups of patients without T2D are required to test the validity of these findings. The aim of the present study was to investigate changes in circulating concentrations of glucose, glucose regulating hormones and peptides, and lipids in patients with morbid obesity without T2D before and six days after SG and RYGB surgery, respectively. Patients without T2D were chosen to avoid heterogeneity in regard to severity of T2D and use of anti-diabetic medications during the study period. The participants underwent a standardized OGTT (75 g glucose), which could be performed on the day before surgery and six days after surgery in a non-diabetic group of patients.

Based on findings in other short-term studies, our hypothesis was that glucose homeostasis would be more strongly affected by RYGB compared to SG six days after surgery.

## Methods

### Participants, study setting and ethics

Patients approved for SG (Voss Hospital, Norway) or RYGB (Haugesund Hospital, Norway) in the period September 2011 to November 2014 were invited to participate in the study. In total, 39 patients were included in the SG group and 27 patients were included in the RYGB group. Of these, 15 patients (six men and nine women) at Voss Hospital and 16 patients (five men and eleven women) at Haugesund Hospital completed the oral glucose tolerance test (OGTT) at both visits. Participants were not required to specify why they withdrew from the study.

Inclusion criteria were body mass index (BMI) 40–45 kg/m^2^, fasting glucose < 7 mmol/L at the medical examination 2–3 months before the surgery, 18–60 years of age, and living in close proximity to either Voss Hospital or Haugesund Hospital. Patients included in the study received the same pre- and post-surgery treatment with regards to medications and diet, and participating patients were exempted from the weight loss program with low calorie diet otherwise recommended at these hospitals before surgery, and had no restrictions on their dietary intake before surgery and before the second study visit. Exclusion criteria were smoking, known T2D, treatment with anti-diabetic medication, and complications during surgery leading to blood transfusion.

The study was designed as an intervention study with quasi-experimental pre-test post-test design, as the criteria for randomization of participants were not met. The study was conducted according to the guidelines laid down in the Declaration of Helsinki and all procedures involving participants were approved by the Norwegian Regional Committee for Medical and Health Research Ethics (approval number: 2010/504). Written informed consent was obtained from all subjects. All data were analyzed anonymously.

### Interventions and study visits

Two types of bariatric surgeries were performed; SG and RYGB. In SG, about 80% of the stomach was removed, leaving a narrow gastric tube along the lesser curvature. During RYGB, a small stomach pouch was created below the cardia and connected to the mid-jejunum; the remainder of the stomach and the proximal intestine is thereby bypassed by nutrient flow.

The total study period was seven days, with examinations the day before surgery and six days after surgery. Examinations were conducted in the morning after an overnight fast. The patients were instructed not to eat or drink anything except water after 22.00 the previous day.

Body height and body weight were measured the day of surgery. Blood samples were collected the day before surgery and six days after surgery, and the staff complied with a strict protocol for pre-analytical sample handling to eliminate differences in sample handling at the two hospitals and over time. At both examinations, glucose tolerance was tested using a standardized OGTT with 75 g glucose dissolved in 100 mL lukewarm water. Patients were instructed to drink the solution over 10 minutes on the day before surgery, as they were expected to need at least 10 minutes to drink this volume six days after surgery. Blood was sampled before (i.e. in fasting condition) and 30 and 120 minutes after drinking the glucose solution.

### Analysis of serum and plasma samples

Blood was collected in VACUETTE® TUBE 4 ml Z Serum Separator Clot Activator tubes (Greiner Bio-one, Austria) for isolation of serum, and BD Vacutainer P800 8.5 ml (Becton, Dickinson and Company, containing K2EDTA and a proprietary cocktail of protease, esterase and dipeptidyl peptidase-IV inhibitors) for isolation of plasma for analyses of GLP-1, glucagon and peptide YY (PYY). Analyses of glucose, insulin, insulin-like growth factor-1 (IGF1), insulin-like growth factor binding protein-3 (IGF-BP3), glucagon, alanine transaminase (ALT), gamma-glutamyltransferase (GGT), alkaline phosphatase (ALP), C-reactive protein (CRP), lipids and total bile acids in serum were performed by routine methods at the Laboratory of Clinical Biochemistry and the Hormone Laboratory at Haukeland University Hospital (Bergen, Norway) and reference values were according to these laboratories. Serum non-esterified fatty acids (NEFA) and albumin were analyzed on the Cobas c111 system (Roche Diagnostics GmbH, Mannheim, Germany) using the NEFA FS kit (DiaSys, Diagnostic Systems GmbH, Germany) and the ALB2 kit for Cobas c111. Plasma concentrations of active GLP-1 were determined using the GLP-1 (active) ELISA kit (EIA-3056, DRG Instruments GmbH, Marburg, Germany). Plasma concentrations of PYY were determined using the Human PYY (total) ELISA kit (EZHPYYT66K, EMD Millipore, Merck KGaA, Darmstadt, Germany). Between-day CVs were between 2.1 and 11% for the methods above. All samples were blinded to the laboratory personnel. The homeostatic model assessment for insulin resistance (HOMA-IR) was calculated from fasting serum glucose and insulin concentrations as glucose [mmol/L] x insulin [pmol/L] divided by 135.

### Outcome measurements

The primary outcome of the present study was changes in fasting and postprandial serum glucose concentrations after RYGB or SG surgery. Secondary outcomes were changes in circulating concentrations of insulin, GLP-1, PYY, glucagon, NEFA and lipids after surgery.

### Statistical analyses

Statistical analyses were conducted using SPSS Statistics version 25 (IBM Corp. IBM SPSS for Windows, Armonk, NY, USA). Subjects that did not complete the study were excluded from statistical analyses. Most data were not normally distributed according to the Shapiro–Wilk test, and non-parametric tests were used to investigate changes within groups (Wilcoxon Signed Ranks Test) and the Mann-Whitney Test was used to compare values between the two groups before and six days after surgery. Changes within the RYGB group and within the SG group were compared using the Mann-Whitney Test. Gender distribution in the groups was compared using Pearson Chi-Square test. Data are expressed as median (25th–75th percentiles). All comparisons were two-sided, and P < 0.05 was considered statistically significant.

## Results

### Patient characteristics

**Figure 1:**
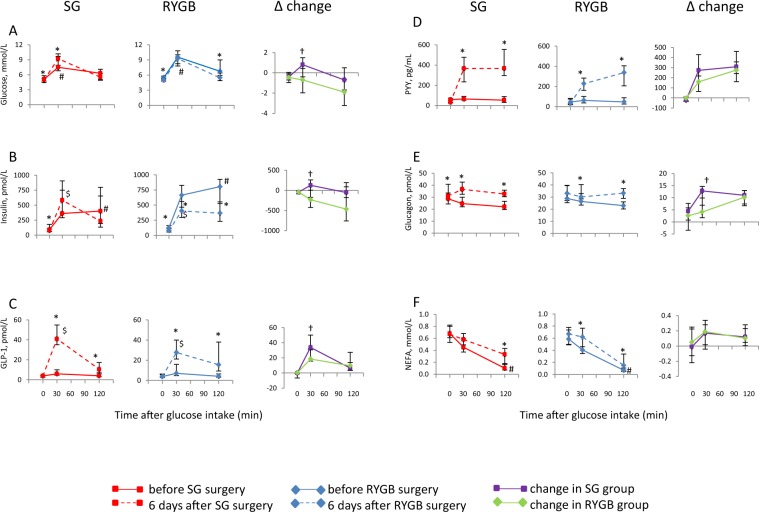
Measurements of glucose, insulin, GLP-1, PYY, glucagon, and NEFA during an oral glucose tolerance test on the day before and six days after SG or RYGB surgery. Comparisons are made within groups for each type of surgery, and between groups for changes in concentrations from pre- to post surgery. Results are presented for N = 15 in the SG group and N = 16 in the RYGB group. Data are presented as median with 25–75 percentiles. *Within group comparisons at the same time point using Wilcoxon Signed Ranks Test ^#^comparison of SG and RYGB values the day before surgery using Mann-Whitney test ^$^comparison of SG and RYGB values six days after surgery using Mann-Whitney test ^†^comparison of changes within group for SG and RYGB values using Mann-Whitney test P values < 0.05 were considered statistically significant.

There were no differences in gender distribution, age and BMI between the SG and RYGB groups of patients (Table 1). Also, there were no differences between the groups with respect to fasting concentrations of any of the measured parameters before surgery, including glucose, insulin, GLP-1, NEFA, PYY, glucagon (Fig. 1A–F), HOMA-IR, IGF1 and IGF-BP3 (Table 2).

**Table 1:** Patient characteristics the day before surgery.

	SG	RYGB	P
Men/women	6/9	5/11	0.61
Age, years	37.4 (35.0–49.9)	40.5 (36.0–53.3)	0.45
BMI, kg/m2	43.1 (41.5–44.6)	42.1 (40.3–44.6)	0.72

Data are presented as median with 25–75 percentiles. No statistically significant differences were seen between the groups at the day before surgery (Mann-Whitney Test). Results are presented for N = 15 in the vertical sleeve gastrectomy (SG) group and N = 16 in the Roux-en-Y gastric bypass (RYGB) group. BMI; Body mass index.

**Table 2:** HOMA-IR and fasting concentrations of IGF1 and IGFB3 in samples collected the day before surgery and six days after surgery.

	SG		P	RYGB		P	SG vs RYGB	
	Day before surgery	Six days after surgery		Day before surgery	Six days after surgery		Day before surgery	Six days after surgery
HOMA-IR	3.5 (2.0–7.8)	2.6 (1.8–4.1)	0.0026	5.3 (3.7–7.7)	3.1 (1.9–4.2)	0.0016	0.25	0.64
IGF1, nmol/L	15.9 (11.6–18.6)	15.3 (6.8–18.9)	0.71	20.0 (13.3–27.4)	14.6 (9.7–17.1)	0.00064	0.089	0.89
IGF-BP3, mg/L	4.2 (3.7–5.6)	3.8 (2.9–4.6)	0.0030	4.8 (3.9–5.5)	3.6 (3.0–4.4)	0.00061	0.59	0.77

Data are presented as median with 25–75 percentiles. Results are presented for N = 15 in the vertical sleeve gastrectomy (SG) group and N = 16 in the Roux-en-Y gastric bypass (RYGB) group.

P; within-group changes are tested using Wilcoxon Signed Ranks Test, Mann-Whitney Test was used to compare the two groups before and six days after surgery. P values < 0.05 were considered statistically significant. Abbreviations: HOMA-IR; homeostatic assessment model for insulin resistance, IGF1; Insulin-like growth factor-1, IGF-BP3; Insulin-like growth factor binding protein-3.

### Fasting glucose- and insulin-related parameters

In patients that underwent SG or RYGB, both fasting glucose and insulin concentrations were lower six days after surgery compared to pre surgery and concomitantly HOMA-IR was also lower, but with no differences between the two groups (Fig. 1A, Table 2). All patients had HOMA-IR above 1.6, which is considered to be the cut-off for normal value^9^. Fasting serum IGF1 concentration was not affected six days after SG but was lower after RYGB, whereas the concentration of its binding protein IGF-BP3 was lower after both procedures, with no difference between SG and RYGB (Table 2).

### Oral glucose tolerance test (OGTT)

An OGTT (75 g glucose) was carried out the day before and six days after surgery. In addition to the clinically relevant 120 min blood sample, we included a blood sample at 30 min. The 120 min glucose and 120 min insulin as well as the 30 min insulin concentrations were lowered by RYGB, whereas SG did not induce this reduction (Fig. 1A,B). Instead SG led to elevated glucose concentration at 30 min. It should be noted that there were some group differences in the OGTT before surgery, with higher 30 min glucose concentrations (P = 0.036) and higher 120 min insulin concentrations (P = 0.0050) in the RYGB group relative to the SG group.

We found no changes in the fasting concentrations of GLP-1 and PYY after the surgical procedures. The postprandial concentrations of GLP-1 and PYY during the OGTT were elevated after both SG and RYGB at 30 min and 120 min relative to before surgery (Fig. 1C,D). Of note, the GLP-1 increment at 30 min was higher in SG than in RYGB, whereas this difference between groups was not evident after 120 min. However, no differences were seen between the groups for fasting and postprandial PYY concentrations. The glucagon concentrations were elevated at both 30 min and 120 min after surgery in both groups, and with a higher elevation at the 30 min time point in the SG group relative to RYGB (Fig. 1E).

Fasting NEFA concentration was not affected by either SG or RYGB. The OGTT revealed higher 120 min NEFA concentrations after surgery in both groups, with no differences between the groups. The 30 min NEFA concentrations were elevated after surgery only in the RYGB group.

### Serum lipids and bile acids

Serum lipid concentrations were similar in the two groups before surgery. After six days the fasting serum concentration of total cholesterol was reduced after RYGB but was not changed after SG, whereas both HDL and LDL cholesterol concentrations were decreased in both groups (Table 3). Fasting triacylglycerol was not changed after surgery within the groups, but the triacylglycerol concentrations were higher six days after SG relative to RYGB. The fasting serum total bile acid concentration was reduced only after RYGB, with no difference between the surgery groups (Table 3).

**Table 3:** Fasting concentrations of lipids in serum taken the day before surgery and six days after surgery.

	SG		P	RYGB		P	SG vs RYGB	
	Day before surgery	Six days after surgery		Day before surgery	Six days after surgery		Day before surgery	Six days after surgery
Total cholesterol, mmol/L	4.6 (3.8–5.3)	4.4 (3.9–4.8)	0.18	5.3 (4.3–5.8)	4.8 (4.0–5.2)	0.010	0.17	0.39
HDL cholesterol, mmol/L	1.0 (0.8–1.2)	0.7 (0.6–1.1)	0.00085	1.1 (0.9–1.2)	0.9 (0.7–1.0)	0.00090	0.52	0.37
LDL cholesterol, mmol/L	3.0 (2.3–3.7)	2.8 (2.3–3.1)	0.038	3.7 (3.1–4.1)	3.3 (2.5–3.6)	0.0060	0.13	0.29
Triacylglycerol, mmol/L	1.40 (1.06–2.18)	1.70 (1.58–2.39)	0.065	1.50 (1.02–2.38)	1.52 (1.31–1.77)	0.64	0.83	0.021
Total bile acids, umol/L	2.0 (0.8–3.0)	2.0 (1.0–2.0)	0.29	2.0 (1.3–3.0)	1.0 (0.8–2.0)	0.013	0.52	0.30

Data are presented as median with 25–75 percentiles. Results are presented for N = 15 in the vertical sleeve gastrectomy (SG) group and N = 16 in the Roux-en-Y gastric bypass (RYGB) group. P; within group changes are tested using Wilcoxon Signed Ranks Test, Mann-Whitney Test was used to compare the two groups before and six days after surgery. P values < 0.05 were considered statistically significant.

### Albumin and markers of organ damage and inflammation

The fasting concentrations of albumin, ALT, GGT, ALP and CRP were similar between the patient groups in serum taken the day before surgery. Serum albumin concentration was marginally increased six days after SG but not after RYGB (Table 4). Serum concentrations of ALT, GGT and CRP were increased six days after SG and RYGB, with no change in ALP concentration. Six days after surgery, no differences were seen between serum concentrations of albumin, GGT and ALP between the two groups, whereas concentrations of ALT and CRP were higher after RYGB compared to after SG.

**Table 4:** Fasting concentrations of albumin and markers of organ damage and inflammation in serum taken the day before surgery and six days after surgery.

	SG		P	RYGB		P	SG vs RYGB	
	Day before surgery	Six days after surgery		Day before surgery	Six days after surgery		Day before surgery	Six days after surgery
Albumin, g/L	45.1 (44.5–46.9)	45.2 (42.7–46.4)	0.036	44.6 (43.6–47.1)	44.5 (43.3–46.5)	0.074	0.43	0.81
ALT, U/L	33 (27–46)	108 (74–133)	0.0019	35 (23–62)	63 (43–101)	0.017	1.00	0.013
GGT, U/L	25 (16–52)	71 (42–102)	0.0045	30 (22–48)	54 (39–65)	0.0032	0.51	0.27
ALP, U/L	68 (61–74)	68 (62–76)	0.53	73 (58–89)	71 (64–85)	0.19	0.35	0.42
CRP, mg/L	4.0 (3.0–6.0)	14.0 (6.0–25.0)	0.0012	6.0 (4.3–10.3)	23.0 (18.0–39.8)	0.00065	0.090	0.034

Data are presented as median with 25–75 percentiles. Results are presented for N = 15 in the vertical sleeve gastrectomy (SG) group and N = 16 in the Roux-en-Y gastric bypass (RYGB) group. ALT; Alanine transaminase, GGT; gamma-Glutamyltransferase, ALP: Alkaline phosphatase, CRP; C-reactive protein. P; within group changes are tested using Wilcoxon Signed Ranks Test Mann-Whitney Test was used to compare the two groups before and six days after surgery. P values < 0.05 were considered statistically significant.

## Discussion

In this study we compared fasting and postprandial circulating concentrations of glucose, hormones and lipids between two groups of non-diabetic adults of both genders with severe obesity undergoing either SG or RYGB. The aim was to gain insight into mechanisms of metabolic changes that occurs shortly after these surgeries. We report that fasting insulin, glucose and HOMA-IR were reduced to a similar degree six days after both SG and RYGB, with no changes in fasting concentrations of GLP-1 and PYY. Postprandial (OGTT, 30 min) serum concentration of glucose was actually higher after SG but was not changed after RYGB, despite increased concentrations of GLP-1 and PYY in both groups at this time point.

The OGTT revealed pronounced effects on postprandial circulating concentrations of glucose, gut peptides and hormones regulating glucose concentrations six days after bariatric surgery, with notable differences between the surgery groups. The reduced postprandial concentrations of glucose and insulin after 120 min suggest that glucose regulation and insulin sensitivity were improved after RYGB, but this was not accompanied with lower postprandial NEFA concentrations as could be anticipated. The increased concentration of glucose and unchanged insulin concentration at 30 min in the SG group suggest that the glucose tolerance was impaired, although no change was seen in 120 min glucose and insulin concentrations after surgery. In line with this, postprandial GLP-1 concentration was higher in the SG group relative to RYGB at the 30 minute time-point during OGTT. Interestingly, this elevation in GLP-1 did not translate into a more pronounced reduction in glucose concentrations in the SG group relative to RYGB, suggesting that elevated GLP-1 and insulin concentrations cannot fully explain the short-term improvement in glucose tolerance after bariatric surgery. In contrast to our findings, Peterli *et al*. reported larger increase in postprandial GLP-1 and insulin after RYGB compared to SG measured 8–10 days postoperatively^8^, and a more pronounced increase in postprandial cholecystokinin after SG compared to RYGB^10^. However, in our patients, both 30 and 120 min postprandial NEFA concentrations were similar after SG and RYGB, whereas the 30 min GLP-1 was higher after SG compared to after RYGB.

The delivery of food to the small intestine will be more rapid after both SG and RYGB procedures, whereas gastric emptying is faster after RYGB compared to SG^11^. After SG, the stomach is restricted and empties more often, whereas after RYGB the patients have a smaller stomach and the food bypasses the whole duodenum and proximal jejunum. The nutrients will reach the distal small intestine more rapidly and get in direct contact with L cells, stimulating the release of GLP-1 which in turn stimulates the secretion of insulin (the incretin effect). Therefore, we cannot rule out the possibility that GLP-1 concentration may have peaked before 30 minutes in the RYGB-patients during the OGTT, although we measured a higher concentration of 30 min postprandial GLP-1 after SG compared to after RYGB.

The postprandial increment of insulin and GLP-1 was higher in the SG group compared to RYGB six days after surgery, and consistent with our results, another recent short-term study demonstrated that postprandial insulin concentrations increased more both two days and 3 weeks after SG but not after RYGB in patients with T2D^12^. This effect changed over time, and after one year follow-up of these patients the postprandial insulin concentrations were highest in the RYGB group^12^. In the same patients, postprandial GLP-1 were increased to a similar degree two days after both SG and RYGB, with higher postprandial GLP-1 concentrations one year after RYGB compared to SG^12^.

In the present study, 30 and 120 min postprandial glucagon concentrations were higher in both groups after surgery compared to pre surgery concentrations, with the largest increase in the SG group. This finding was unexpected, as both glucose^13^ and GLP-1^14^ inhibit glucagon release, and since the 30 min postprandial concentrations of glucose was increased after SG and the GLP-1 concentrations after 30 and 120 min were increased after SG and RYGB. Others have also found that postprandial glucagon secretion was not inhibited by meals or glucose after gastric bypass despite higher GLP-1 secretion in patients^15–17^ or in rats after gastric sleeve or gastric bypass surgeries^18^. This suggest that the SG patients may have an impaired inhibitory effect of glucose on glucagon secretion (alpha-cell resistance), which has been observed in an alpha-cell model of insulin resistence^13^.

The effect of bariatric surgery on lipid metabolism has been investigated in only a few studies, some showing that circulating lipid concentrations are improved 6–24 months after both SG and RYGB^19–21^, probably as a result of surgery-induced weight loss. After six days in our study, SG and RYGB did not affect serum triacylglycerol concentration, but both surgeries resulted in lower serum concentrations of HDL and LDL cholesterol, whereas total cholesterol and total bile acids concentrations were decreased only after RYGB. These divergent effects on lipids may be explained by malabsorption after RYGB as total bile acid was lower after surgery in this group, or lower energy intake as serum IGF1 was decreased after RYGB. Surgery-induced stress likely explains the increased CRP concentrations after both SG and RYGB, and the higher CRP after RYGB surgery may be because this is a more invasive procedure compared to SG. As patients were in the acute-phase when post-surgical samples were collected, as was evident by the increased CRP concentration in both groups, serum amyloid A released from liver will replace apolipoprotein A-1 in HDL and is also distributed in the LDL fraction^22,23^. Declines in LDL and HDL cholesterol concentrations have been reported in the acute-phase after surgery^23^. The serum concentration of ALT, a marker of hepatic function including inflammation, was also increased after both surgeries, but to a higher concentration after SG compared to RYGB which is most due to a rougher manipulation of the liver during the SG procedure.

This study has some strengths and limitations. All blood samples from both hospitals were analyzed simultaneously by the same laboratory to minimize technical variability. We included only non-diabetic patients to avoid heterogeneity in regard to severity of T2D and use of different types, doses and combinations of anti-diabetic medications. Both men and women were included, and the staff complied with a strict protocol for pre-analytical sample handling to ensure high sample quality. Patients scheduled for RYGB may have had poorer postprandial glucose regulation compared to those scheduled for SG, since the RYGB group had higher concentrations of postprandial insulin before surgery. Since the SG and RYGB groups had similar HOMA-IR level before surgery, this suggests comparable insulin sensitivity in the fasting state in these groups. In the present study we show fasting bile acid concentrations before and after bariatric surgery. Future studies should investigate the effect of bariatric surgery on concentrations of individual bile acids after intake of a mixed meal to provide more information about the glucose metabolism. Inclusion of a larger number of study participants may reveal additional differences in fasting glucose, insulin or other variables, although we found several differences in postprandial responses. Further, due to limited staff resources we were able to sample blood only at two postprandial time points, and hence could not calculate area under the curve values for glucose, hormones and lipids.

## Conclusions

Our results show that SG and RYGB similarly affected fasting glucose homeostasis six days after surgery in non-diabetic patients, with lower concentrations of glucose and insulin as well as lower HOMA-IR. However, postprandial glucose regulation and insulin sensitivity seem to be improved six days after RYGB but not after SG, possibly due to enhanced insulin sensitivity rather than augmented insulin release. The present study provides new data on the short-term effects of vertical SG and RYGB, and expands the knowledge on the mechanisms involved shortly after bariatric surgery. The patients in the present study were non-diabetics, with HOMA-IR above normal level thus displaying reduced insulin sensitivity. Future studies should investigate the short term effects of SG and RYGB in T2D patients, to evaluate whether these patients will experience similar metabolic effects shortly after surgery.

## Data Availability

Data will be made available upon reasonable request.
